# What are families most grateful for after receiving palliative care? Content analysis of written documents received: a chance to improve the quality of care

**DOI:** 10.1186/s12904-017-0229-5

**Published:** 2017-09-06

**Authors:** María Aparicio, Carlos Centeno, José Miguel Carrasco, Antonio Barbosa, María Arantzamendi

**Affiliations:** 1 St John’s Hospice, London, UK; 2 Universidad de Navarra, ICS, ATLANTES, Campus Universitario, 31080 Pamplona, España; 3 Clínica Universidad de Navarra, Departamento de Medicina Paliativa, Avenidad Pío XII, 31080 Pamplona, España; 4 IdiSNA, Instituto de Investigación Sanitaria de Navarra, Grupo: Medicina paliativa, Pamplona, España; 5 Centre for Bioethics, Faculty of Medicine, University of Lisboa, Lisbon, Portugal

**Keywords:** Quality of care, Outcomes, Palliative, Families, Qualitative, Home-based palliative care, Relationship

## Abstract

**Background:**

Family members are involved in the care of palliative patients at home and therefore, should be viewed as important sources of information to help clinicians better understand the quality palliative care service patients receive. The objective of the study was to analyse what is valued most by family carers undergoing bereavement of a palliative care home service in order to identify factors of quality of care.

**Methods:**

Qualitative exploratory study based on documentary analysis. Content analysis of 77 gratitude documents received over 8 years by a palliative home service in Odivelas, near Lisbon (Portugal) was undertaken, through an inductive approach and using investigator triangulation. Frequency of distinct categories was quantitatively defined.

**Results:**

Three different content categories emerged from the analysis: a) Recognition of the care received and the value of particular aspects of care within recognised difficult situations included aspects such as kindness, listening, attention to the family, empathy, closeness, affection and the therapeutic relationships established (63/77 documents); b) Family recognition of the achievements of the palliative care team (29/77) indicated as relief from suffering for the patient and family, opportunity of dying at home, help in facing difficult situations, improvement in quality of life and wellbeing, and feeling of serenity during bereavement; c) Messages of support (45/77) related to the need of resources provided. The relational component emerges as an underlying key aspect of family carers’ experience with palliative care home service.

**Conclusion:**

Family carers show spontaneous gratitude for the professionalism and humanity found in palliative care. The relational component of care emerges as key to achieve a high quality care experience of palliative care homes service, and could be one indicator of quality of palliative care.

## Background

Palliative care considers the patient and the family as the focus of care and includes the bereavement period. The family members provide and receive care as the illness of the patient influences their own lives and roles. In this sense, family carers’ perspectives help us to understand palliative care as the families witness and also take part in patients’ care. The family values the care that the patient receives [1], so they should be included in trying to identify factors of quality of care [2–6]. Results of studies suggest that a family carer’s mood and grief can influence in his/her assessment of service provision. Despite this, family carers have been identified as a valid and vital source of information [7, 8]. Family carers, are those with close social and /or emotional, but not necessarily blood relations to the patient; they can be important sources of information [9], and they are even more relevant when considering situations in which the ill person has been cared for at home, where these carers play, arguably, the most essential roles.

Family carers face multiple challenges assuming a carer role and often may feel unprepared [10–13]. Palliative care home services have been developed to support patients and their families at home during advanced and terminal disease, and care can be delivered in a variety of different ways (i.e: home care teams attached to inpatient hospices, community hospice care agencies, hospital-based community support teams and hospital at home services).

It is important to assess the quality of care of palliative care home services in terms of what quality of care means for patients and their families [5]. In order to avoid reductionism, qualitative methods have been proposed [14] showing people’s views within a wider context [15] and exploring more than just “structure”, “process” and “outcomes” of quality of care framework [16, 17]. Inductive approaches can inform alternative approaches to measuring the quality of palliative care services. They can promote, assessing all dimensions of care, as the Council of Europe encouraged [18], especially since tradicional health indicators [17] such as death or recovery rates, or even place of death [19] might not be appropriate as quality indicators in palliative care. It is now recognised that quality of care evaluation is moving away from performance measurements, uniformity of services and standardisation of processes, to providing a greater focus on care [20], covering the full range of aspects needed for quality end-of life care [21].

In fact, there are increasing studies reporting on how palliative care home services are valued by patients, families and health professionals. Studies involving quantitative approaches have generally shown positive impact of these services. A Cochrane review evaluating impact of palliative care home services including 23 studies with patients with different advanced conditions showed significant beneficial effects of the service on reducing symptom burden for patients; although there was no effect on caregiver grief [22]. Value of these services has been reported also on aspects such as: advanced directive completion, site of death, symptom severity over time, program satisfaction [23], and hospice referral and average length of stay [24]. More recent studies using inductive approaches provide a complementary view of these services’ value for family carers. Twenty interviewed family carers’ highlighted aspects such as experiencing a valued presence and not feeling alone and vulnerable, enabling brief periods of rest, and supporting their normal life routines and carrying out different responsibilities [25]. Another study elicited patients (*n* = 16) and family carers’ (*n* = 5) experiences with hospice at home care. Embracing holistic care was the overall key message. Participants value talking about difficult discussions such as death and dying, feel that patient and family carer situations are better understand and their needs and wishes remain central to their care. They also valued the expertise of nurses as they ensured symptom control and guided them in navigating them towards appropriate services. Participants also reported the importance of promoting choices and enabling them to meet their needs at home, preventing unwanted hospital admissions [26]. Family carers express appreciation when health professionals are competent and flexible, and have appropriate communication and carer involvement. They enjoy and desire good relationships with health professionals [27]. Other studies provide a more complex view of home care and raise the difficulties that family carers encounter in accommodating a relative being cared for at home, such as moving in of important objects needed at home and the painful reminder these objects reflect when the loved one passes away, as well as the changes in identities as they assume new roles as carers versus their previous role as wives or husbands [28].

A better understanding of what families’ value most from palliative care home services can be the first step to identify key aspects of quality of these services and promote aspects of excellent care. Considering family carers’ spontaneous letters and notes about their experience with the home palliative care provides an innovative way of assessing this type of service. The letters transmit what families carry with them during the bereavement process and can be considered as having a special value as writing letters is a self-initiated act and is open-ended in terms of its contents (e.g. may range from thankful to aggressive). Grateful family carers can be a source of information about what aspects are valued positively and can direct palliative care home services towards the care that patients ultimately want to receive.

A series of letters of gratitude have been identified by the authors in palliative care services in many different places as far apart as Cape Town (South Africa), Stockholm (Sweden) or Las Palmas de Gran Canaria (Spain). In palliative care, gratitude from patients or their family carers constitutes a reality that often occurs during the mourning process of families who must fully face the intensity of palliative care. A previous study of our group on a different collection of letters gave us the opportunity to explore and reflect on the deep experiences of the family in palliative care [29]. Here, we are using a different focus looking at their vision of what kind of quality of care was received. Considering that the information was provided voluntarily, it may contribute to an increased awareness of family carers’ views on the services received. The current study is especially relevant as there is a lack of studies on opinions provided spontaneously by users of health services. This study’s objective was to analyse what is valued most by grateful family carers undergoing the bereavement process of a palliative care homes service, in order to identify factors of quality of care.

## Methods

A qualitative exploratory study was conducted to learn from family carers’ spontaneous writings about their experiences with the palliative care home service using an inductive approach.

### Study context

The Ongoing Integrated Care Team of Odivelas (ECCIO) was a pioneer team in the area of health care at home in Portugal. It gave assistance to the population enrolled in the primary care center of Odivelas (120.365 people were enrolled there); situated at the north outskirt of Lisbon. The population attended by the team was composed of mainly elderly persons with limited economic resources and education.

The ECCIO was developed in response to the home care needs of an increasingly aging and dependent population. The ECCIO was a multidisciplinary team that was made up of a doctor, nurses, a social worker, a psychologist and other workers, all with specific training and over 8 years of palliative care experience as a group. One thousand patients were cared for each year.

Patient admition criteria included: being a resident in the geographic area of Odivelas and enrolled in the primary care center, having an advanced disease leading to physical dependence, and having at least one carer at home.

The goal of the team was to provide direct health care at home in the field of promotion, treatment, rehabilitation and palliation; providing support and information to family; monitoring patient care, and coordinating between the different health professionals and other institutions. It was the first team providing health care 24 h a day, 7 days a week, to cancer and non-cancer patients. The team provided scheduled health care to patients from 8 am to 8 pm every day; and emergency care around the clock being a nurse the contact.

In case there was need for personal hygiene, food hygiene or housing, social support agencies were contacted, including a combination of public and private agencies who provided nursing assistants to help those with special needs.

### Population and sample

In the period under study, the ECCIO attended over 130 end-of-life events, 85% of whom passed away in their own homes. The majority of the patients had cancer (with many symptoms) and most of the rest were brain stroke victims. Most of the patients were older than 65 years.

Primary family carers were mostly wives, above 65 years old, generally from low to low-middle class backgrounds with little education. But they often had the support of other family members such as son/daughters or neighbors, and therefore, there was strong social support. These details are extracted from the annual activity report of the service to which MAP had access with the permission of the service.

The sample includes all written gratitude documents sent by patients’ family carers to the ECCIO team since its founding in 1998 until the year 2006, expressing their gratitude to the health professionals for the care provided. Letters from families on personal matters to individual members of the team were excluded. The authors actively looked for complaint letters during the study period, but none were found.

### Data collection and analysis

The gratitude letters received by the palliative care home service were filed by the team, independently of their length. The documents were sent to the ECCIO and some were published in local newspapers, and a copy send to the ECCIO. The original documents were copied and anonymised. The original quotes were in Portuguese and have been translated from the original. The documents were written by the family carer. During the analysis of the data no repeated surnames were identified on the documents. On the basis of this and that often the person writing the letter said that he transmited gratitude not on his/her own name –referring to the kinship with the deceased- but on behalf of the whole family, we assume that each document belongs to a different family, but there may be exceptionally more than one letter from a family.

A document analysis was conducted, which is a systematic procedure for reviewing or evaluating documents [30]. As in any other analytical method in qualitative research, it involves examination and interpretation to elicit meaning and gain understanding [31]. The letters had been developed without researcher’s intervention, so we used them to see what family carers mention spontaneously without any external intervention. Documentary analysis have been previously used in other contexts ie. service use among families living in poor urban communities [32].

The whole documents were used for the analysis. It was expected that the letters will reflect the positive experiences of the work that the home team of palliative care service conducted. No predefined list of possible key categories was used. It was thought that categories different from previous work could emerge as the setting was different. An inductive approach was used to explore the key aspects that family carers highlighted from their experiences that were embedded in the letters. The content analysis carried out was based on Burnard’s guidelines [33]. He describes 14 stages of the qualitative analysis process, including the writing. He proposes immersion in the data, reading again and writing down as many headings as necessary to describe all aspects of the content, reviewing list of categories and grouping together similar categories and removing repetitious or very similar headings. Several colleagues analysed the documents independently and lists of categories were discussed and adjusted as necessary. Data was re-read alongside the finally agreed list of categories and sub-headings to establish the degree to which categories covered all aspects of the data. Each data set was coded and all items of within each code were collected and all the codes filed together for direct reference when writing up the findings and selecting examples from the data.

### Rigour

Information is provided about the type of document and the possitive nature of the documents. All the documents were analysed in full. All this promoted confirmability. The letters published in newspapers were often cut out and sent to the palliative care home service, so that it could be argued that it really transmited patients’ families views and that their messages were not altered at the point of publication.

Reliability was enhanced through investigator triangulation [34]. Three researchers read the original unidentified texts, performed an independent analysis and agreed on the descriptive categories, and subcategories, to be used. All the information in the documents was verified as having been suitably coded and represented in the categories. These categories were finally defined by mean of discussion and consensus among several researchers not involved in the care of the patients. During the entire analytical process, reflexivity was encouraged through ongoing debate among the researchers [35].

As well as the content analysis, a count of the number of appearances of each sub-category in the documents was kept in order to identify the frequency with which the categories are referred to in addition to its discursive value. Irrespective of their frequency, all aspects mentioned by family carers in the documents were considered when developing the categories. The quotes are examples of the categories.

Transferability was promoted providing a rich description of the context and the data, and the discussion of the result with what is known so far [36].

### Approvals and ethic considerations

At that time the approval and assessment process of studies was performed by the centre management board. The project and its procedures were reviewed, and written approval was obtained by the management department of the health centre that ECCIO belongs. The project did not involve intervention of human subjects so this approval was considered sufficient by the health centre. Confidentiality was assured and there are no identifiable details within the manuscript. In fact the documents have been identified by number and initials according to the type of document.

## Results

Seventy-eight documents were collected by the ECCIO in the 8 years following its founding; after applying the exclusion criteria 77 were selected for inclusion in this study (no letters of complaint were found). The documents analysed came in different formats: 27 post cards (PCS), 24 letters sent to the team by post (PL), 11 letters to the Health Centre’s Management Department (DL), 8 Christmas or Easter greetings cards (CEC), and 7 letters published in local newspapers (NL). Whilst only 17 documents refer to the first six years of activity, 21 refer to the year 2004, 14 to 2005 and 25 to 2006. The researchers were not able to link the letters to the patients cases. The analysis of this material led to the definition of 3 categories and 18 sub-categories (Table 1) related to aspects of the care received: a) Recognition of the care received and the value of particular aspects of care within recognised difficult situations (in 63/77 documents; seven sub-categories); b) Family recognition of the achievements of the palliative care team (in 29/77 documents; six sub-categories) and; c) Messages of support (in 45/77 documents; five sub-categories).

**Table 1:** Categories, sub-categories and their frequences of identification in the total number of 77 selected documents

Category		Sub-category	
Title	Number of documents where categories appear	Title	Number of documents where sub-categories appear
Recognition of the care received and value of particular aspects of care within recognised difficult situations	63	• Positive overall evaluation	34
		• AAA+ for the team	9
		• Positive evaluation compared to other units	4
		• Positive evaluation of specific aspects of care	64
		• Positive evaluation of staff humanity	30
		• Positive evaluation of professional competence	25
		• Intimate and emotional farewell as relationship is valued	15
Family recognition of the achievements of the palliative care team	29	• Relief of suffering	24
		• Dying at home	10
		• Improving the family capacity to cope	9
		• Quality of life and well-being	9
		• A calm end-of-life	3
		• Feeling of serenity during bereavement	6
Messages of support	45	• Encouraging the team	13
		• Greetings	12
		• Blessings	31
		• Desire to see more teams	9
		• Offers of cooperation	3

### Recognition of the care received and value of particular aspects of care within recognised difficult situations

Documents provide information of the global evaluation that family carers, probably unconsciously, carry out. Family carers’ primary objective was to thank the health professionals for what they valued most from the care received; but it could be argued that this indirectly entails an evaluation of the care itself.

Family carers transmited gratitude in situations that they described as difficult, painful, complex or crucial. But in the midst of these circumstances they offer their written thanks as they identify key aspects of the care provided.

Many documents contain a *positive overall evaluation* by the family carer of the work undertaken, and use adjectives that often transmited beyond a ‘strictly’ professional contribution. The work was considered to be indispensable (6/77), exemplary (5/77) or positive in different ways (23/77), as well as noble, difficult, dignifying or unforgettable.

*“We can not fail to reinforce the great merit of your work…that during this time we lived and that marked us in this critical time of our lives” (PL37).*

*“Because we believe that we must publicly acknowledge these facts, we inform that we send a letter with identical content to the Ministry of Health, as well as to the Health Subregion of Lisbon, so that they know the essential and valuable service that you provide, in the field, to patients and relatives” (PL53).*

*“All of you undertake a difficult task, so noble, so necessary and useful for patients and families!” (PL18).*

Nine documents refer not only to positive perspectives but also speak about the *highest possible quality* with regards to the team’s actions, with words such as fabuolous, marvellous, spectacular, or formidable:

*“A five star team” (PCS31).*

*“To all of you “Well done” for the splendid work you do” (PC17).*

For 4 families, the positive view inevitably led to the *comparison with other services*.

*“At times, hospitals do not offer the best conditions needed to work with them and what often happens is that they are left unattended, even by competent staff, as in hospitals, there are no friendly words or kindness for them, the opposite of home care” (PL67).*

Beside overall positive comments, nearly all the documents highlight *specific aspects of care* that were appreciated. It is specified in the documents that the attention provided was extensive to the needs of the family carers. Family carers felt included in care. Around half of the families refered to the care they themselves had received and for the support given to them during their loved ones’ illness but also after the patient’s death.

*“…[thank you for] helping the ill and not only the ill, but also for helping family members who are depressed and unaware of how to treat a patient” (DL15).*

*“…as well as the availability of the whole team to support the family during the illness and after passing away” (PL44).*

They also mention the ongoing care during the illness (25/77), twenty-four hour attention (11/77), and the round-the-clock presence in the patients’ homes.

*“Day and night, this team was so competent and helped me, my daughter and my loved one (…)” (DL11).*

*“Somebody who worries about patients 24 hours a day and who is fully available, always with a smile and a friendly, comforting word, (…)” (PL26).*

Other categories included in the documents are reflections on *the care provided for in terms of the humanity and competence.* Aspects that, although referred to separately, are key in the patient-health professional relationship. Most of the families acknowledged the *“humanity” of the care given*, which was expressed as kindness, humanity, friendship, empathy, humane gestures, or love.

“*All the kindness which you showed, your patience at so many times, the friendly smiles and gestures which so often provided comfort! We are so grateful!” (S54).*

*“… praise your work, which is carried out with the greatest humanity” (CEC8).*

*“... overcoming relationships that could be strictly professional, showing affection and friendship” (DL 78).*

References to *“professionalism”* appears frecuently in the documents, including factors such as availability, competence, professionalism, effort, knowledge, honesty, and the ability to listen.

*“…our acknowledgment and thanks to the whole team (…), for the competence, professionalism… which was shown and applied, during the final days of our beloved relative” (PL18).*

*“I especially [thank you for your] availability, the speed with which you attended our phone calls or personal requests for help when required” (PL65).*

*“It is not easy in the present day to find this availability and dedication” (PL62).*

Apart from the humanity and competence that families highlight, which are key in the patient-health professional relationship. The relationship established, and the importance of it for families is reflected in an intimate and emotional farewell in 15 letters, reinforcing the idea of the importance of the relationship experienced during the caring process:

*“Anyway, I just know, I do not know how to thank ... for many words I use I will not be able to convey my appreciation for everything you did for my “so beautiful, loved and complete” family. I can only say that you will always remain in our hearts” (PL33).*

*“A great embrace of friendship” (CEC25).*

### Family recognition of the achievements of the palliative care team

The different results achieved by the palliative care home service are mentioned in a significant number of letters, reflecting the extent to which main palliative care goals were achieved [33]. Family carers use expressions such as, “you make us or you showed me…” to transmit that merit is due to the palliative care home service. These achievements can be considered as outcomes identified by family carers that they attribute to the palliative care team.

Some family carers specified that the team helped to *relieve or lessen patient and family suffering* (24/77).

*"you contributed decisively, in order to mitigate the physical and emotional difficulties that my husband ... passed" (PL66).*

*“In this sense, wife, daughters, sons-in-law and grandchildren recognize your contribution on relieving his pain and all his suffering” (PL61).*

*“With your ability and sense of solidarity, you managed to make us feel less alone in the most painful moments which we had and I am sure, helped lessen my father’s suffering” (CEC39).*

They refer to the health professional’s acompaniment that reduces loneliness and suffering, which requires a therapeutic relationship, as the use of only medicines neither decreases loneliness nor the different types of suffering*.*

In some documents, family carers used different kinds of trascendental expressions such as, “serenity in our hearts” or “love when nothing else is left”, that transmit relief of a more spiritual suffering (7/77).

*“The serenity we could feel in our hearts due to your attention was a wonderful thing” (PL33).*

*“[you allowed me] to give the greatest possible love to someone who had nothing else left” (PL51).*

Others referred to the achievement of their loved ones *dying at home* (10/77).

*“It was only with your help that it was possible for my husband to stay at home” (PL41).*

Some family carers also transmitted that they felt empowered in *improving the family’s capacity to face the situation* (9/77).

*“What we learned with you will stay with us forever, in our memory and in our heart” (PCS12).*

*“… you have shown me how to face up to the illness and to death in another way” (PL62).*

Family carers also mentioned *achieving quality of life or well-being* for their loved one as something that the palliative care home service made possible (9/77).

*“you have provided a better quality of life, which if possible would be free of pain” (DL78).*

*“…you contributed over these years to the well-being of my beloved mother” (PCS13).*

*A calm end of life* (3/77) is also something that family carers valued:

*“…he passed away very peacefully, as if entering a deep sleep” (DL78).*

Family carers in 6 letters also transmited *achieving feeling of serenity or a calm feeling during bereavement* due to the contribution of the palliative care home service.

*“I am aware that I have done everything to ensure he had the least suffering possible during the period of illness, and then in death. But this situation was largely achieved only by your extraordinary support ...” (PL62).*

*“You let me prevent further suffering at the most critical moments and at the same time, give more love to somebody who had little else left. So important that it makes me feel calm of spirit, comforted in bereavement…” (PL51).*

### Messages of support

The documents contain messages of support, encouragement and suggestions about creating the same type of service in the rest of the country, and even offers of help. They transmit perceptions about the need for the type of care they received. In many cases family carers write messages to support their continuity, as if they were *encouraging the team to continue with the good work* (e.g., “Carry on that way!” 13/77).

*“…I wish you strength and courage and my most sincere congratulations so that you manage to continue down that road, until the end [of a persons life]” (PL69).*

Christmas and Easter are special dates when people exchange greetings and good wishes with appreciated people. Some family carers followed this practice and sent *greeting cards* (12/77) in these special dates to expresss their gratitude for the care received.

*“…my most sincere greetings to all of you” (PL66).*

*“A Merry Christmas and a Happy New Year” (PC6).*

In almost half of the letters (31/77) there are texts of *blessings* transmitted to the staff during the time in which family carers are mourning.

*“For all of you, our blessings” (NL19).*

Nine messages were found in which the family carers express their *desires to see more teams* with similar characteristics set up in other parts of the country in order to provide care for those in need.

*“It seems incredible that it is possible that there are not more “marvellous teams” all around the country, to provide comfort and a certain degree of quality of life to patients who so need your smile and professionalism” (PL33).*

And three family carers *offered to co-operate* and help the team in whatever way the are able:

*“(…)I know I can help your team in some way. As a Nurse? It is a little late for that. A voluntary support for families, who knows! (…) I repeat that I am at your disposal for whatever you need, even if that just means driving” (PL50).*

## Discussion

This study shows that some family carers highly value their experience with the palliative home care team. They specify the contribution that the team made from their perspectives and congratulate and encourage health professionals to continue working in the same manner with other families. All this is reported with adjectives and certain familiarity in the language that recalls a good patient/family-health professional relationship. The relational component acquires more relevance when the messages of support are considered. Family carers transmit a need for reciprocity, to provide something back, which happens within any good relationship.

It is noteworthy that within the positivism of the documents, there is also recognition of the difficulties of the situations faced caring for loved ones at home, which is also reported in a comprehensive review of the literature on home-based care giving at end of life [37].

Family carers transmit an overall positive view of the services received, which is consistent with the Cochrane reviews on palliative care home services [22]. However, the wordings used such as ‘from the bottom of our hearts’, recall deep interpersonal positive experiences. Within the overall satisfactory comments family carers highlight the humanity and professionality of the palliative home care team professionals, a combination that Cicely Saunders [38], pioneer in palliative care, always considered essential and advocated for in an interpersonal relationship between the ill person and the health professional. She believed that the person of the health professional could be sometimes the best medicine. The combination of professionalism and humanism echoes one of the statements of a review on palliative care home services that affirms that it is important to see improved quality of life and enhancement of human dignity as a result of palliative care home services [39]. One particular aspect, the patient’s perception of dignity, has been shown to be influenced by the type of interaction that occurs between the ill person and others [40].

Family carers spontaneously specify what the palliative care home service has achieved and attribute all merit to the team. Lessening patient and family carers suffering through health professionals’ accompaniment is one of them, as families felt helped and felted a sense of solidarity. Literature about family carers says that carers have different support needs because of the risks associated with increased burden, depression or physical tiredness [41, 42]. In fact, studies about hospice at home services point out that family carers highly valued having brief periods away from the ill person. Health professionals enabled family carers to have brief periods away for the ill person, providing some respite and supporting life outside of the care-giver role [25]. However, in the current study, this service was not available but the carers still felt supported. They felt cared by the health professionals but also felt that they had enhanced their capacity to face various difficult situations. Thus, it could be argued that they felt as though they were participants along the process, which requires building relationships.

The fact that their loved ones were able to die at home was another achievement mentioned by family carers. However, it was not mentioned so often as expected based on the literature, where it has been considered as a key quality care indicator. It might be that, as Pollock [19] suggests emphasis on place of death overlooks other aspects of care that may be important for family carers. Considering that among the factors influencing place of death are the illness, the individual, and the environment (healthcare input and social support), it might be worth considering other indicators. Especially if it is taken into account that there is growing evidence that care at home may cause tensions and that social relationships shape decisions about place of care [28]. This raises the question whether social relationships should be taken into account when assessing quality of care. In fact, in another study about family carers’ experiences about institutionalized palliative care services (hospice [29], inpatient unit [43]), families highlighted the importance of the warm environments that they experienced. The type of environment was not mentioned in the current study, probably because it was in patients’ homes.

Improving patients’ quality of life and well-being is another achievement that family carers mention. This is in line with a Cochrane review that examines home palliative care services measuring outcomes for patients and their caregivers such as symptom control, quality of life, caregiver distress and satisfaction with care [22]. But unlike other qualitative studies, in the documents, family carers do not mention often specific symptoms, except for pain. They tend to refer more to the alleviation of suffering in all of its forms, which shows a broader concept of suffering than physical pain, requiring a more holistic approach. The relevance of holism emerges also in one study about patients’ and families’ experiences of a hospice home care service. There, this approach was identified as having a positive impact on emotional, psychological, social, and physical well-being [26].

Considering all the achievements highlighted by the family carer, there is an underlying idea, that the relational aspect is key between the patient/family and the health professional. This is in accordance with the statement that quality in end-of-life care has more to do with relationships and communication between attending health professionals and the seriously ill patient and family than patient autonomy [5]. In fact, from the perspective of patients, families, and health professionals, caring for the terminally ill patient as a whole person is essential. However, despite family carers’ emphasis on more relational aspects, these have received scarce consideration among quality indicators.

A limitation of the study is that using documents entails that there is only information on what is written, which varies in length and depth, and there is no possibility in clarifying or extending the information. But the available length of the documents also promotes prioritising and highlighting the most valuable and important aspects. The documents were produced for purposes other than research, which might be considered a limitation, but also a strength as there was no intervention [31]. The documents only include positive perspectives as no negative documents were found, which might be because they were no negative experiences or because the negative experiences were sent to other regulatory bodies. Having included only the thankful family carers’ opinion is a limitation. There may be different perspectives, of those who send complaints or do not write documents, which might suggest other general dimensions that they might like to have included in order to assess the quality of palliative care received.

As mentioned earlier we did not have predefined list of possible key categories but not having reflected a priori on our pre-existing ideas is a limitation. The use of researcher triangulation may have decreased possible influence of our pre-existing unconscious ideas on data analysis. Future studies would benefit from identifying and including family carers with a range of experiences with different home-based palliative care services.

There is interest in knowing users’ views on the services received. Inductive approaches, like the one used in the current study are recognized as valuable. But rarely have comments -made by the users about the care received, without being asked about explicitily, been analyzed. Considering family carers’ spontaneously written documents can provide new insights into quality of care, as they provide different points of view and are sources of information on aspects of care that are important to them.

Elements of the relational component of caring for terminally ill patients and their family carers at home should be included. It could be argued that some aspects are considered when including in quality care questionnaires items such as bedside manner, common courtesy, and way of talking [44]. Communication issues could be considered as part of the relational aspect, and that is what has been considered within the quality indicators but focussing mainly on informing and decision making [44, 45]. More recently, aspects such as developing rapport, addressing expectations and listening actively have been included [43]. But family carers’ descriptions point out that there is more beyond the human interaction between the patient/family and the health professional. Family carers highlight that palliative care home services have helped to improve their capacity to cope and learn to face various situations. Family carers need to feel reassured that the professionals would support them when needed in order to cope with caring for their loved ones at home [46]. Therefore, grateful family carers’ comments could be considered when developing quality indicators about the outcome of care in relation to work with the family carers. All of this is in accordance with the idea of implementing quality indicators that are reflective of the scope of care [47].

If patient and family carer want to be involved in the care process, taking into account aspects that they value most is a good starting point. It is essential to have in mind the importance of the health professional-patient/family carer relationship. This also has implications for health professionals’ practice, as often the pressure and limited resources, specially on a economic crisis period, can lead to focus on conducting tasks more than on caring for the person who is ill and his family.

Further research should include more palliative care units and explore opinions of those family carers who send complaints, or who do not write documents, to consider other general dimensions that they might like to have included in order to assesss the quality of palliative care received. It would also be interesting to assess if the positive documents sent by bereaved family carers have any repercussion on the quality of care that health professionals provide or in their motivations to continue caring for these type of patients.

## Conclusion

Knowing family carers’ perception and understanding their experiences is a chance to enhance how to be helpful to patients and family members, and allows palliative care professionals to identify positive aspects of their care in order to improve the assistance provided to patients and family carers. Despite the sadness of their loss, comments from family carers provide valuable input about their points of view of the scope of care. Family carers highlight the importance of key aspects that require a close relationship between health professionals and themselves, suggesting that this component or aspect could be used as a quality indicators of care.

## Abbreviations

CEC: Christmas or Easter greeting cards
DL: Letters sent to the Health Centre’s Management Department
ECCIO: Ongoing Integrated Care Team of Odivelas
NL: Letters published in local newspapers
PCS: Post cards
PL: Letters
